# Changes in blood physiological and biochemical parameters and intestinal flora in newborn horses and mares with angular limb deformities

**DOI:** 10.3389/fvets.2024.1503117

**Published:** 2024-11-26

**Authors:** Yuhui Ma, Yigang Liu, Hai Li, Kailun Yang, Gang Yao

**Affiliations:** ^1^College of Animal Science and Technology, Xinjiang Agricultural University, Urumqi, China; ^2^College of Veterinary Medicine, Xinjiang Agricultural University, Urumqi, China; ^3^Xinjiang Zhaosu County Xiyu Horse Industry Co., Ltd., Zhaosu, China

**Keywords:** angular limb deformities, equine, intestinal flora, skeleton, metabolism

## Abstract

**Introduction:**

Angular limb deformities (ALDs) are a common skeletal development disorder in newborn foals. This condition affects the growth and development of foals and severely impacts their future athletic performance and economic value, causing significant financial losses to the horse industry. Placentitis, metritis, and severe metabolic diseases during mare pregnancy are significant causes of ALDs in newborn foals. It has been established that intestinal flora disorders can easily lead to inflammatory and metabolic diseases in the host. However, the incidence of ALDs in foals in Zhaosu County, Xinjiang, a key production area of China's horse industry, remains unclear. Additionally, the relationship between functional changes in foals with ALDs and their mares and changes in their intestinal flora is not well-understood.

**Methods:**

This study investigated the status of ALD in newborn foals through clinical observation and imaging examinations. In addition, molecular biological methods were applied to examine the effects of ALDs foals and their mares on physiological and biochemical markers and gut microbiota.

**Results:**

The results showed that the incidence of ALD in Zhaosu area of China was 4.13%. In addition, by comparing and correlating the physiological and biochemical indicators and intestinal flora of foals and mares with ALD with those of healthy horses, it was found that foals and mares with ALD may promote the occurrence and development of the disease through the “blood marker changes-intestinal flora-ALDs” axis. In addition, by comparing the physiological and biochemical indicators and intestinal flora of foals and mares with ALD with the intestinal flora of healthy horses, it was found that the physiological and biochemical indicators and intestinal flora structure and metabolic pathways of foals and mares with ALD had significant changes.

**Discussion:**

The diversity, species composition, and function of the intestinal flora of ALDs and their mares were significantly altered. These findings provide a scientific basis for understanding the etiology of ALDs in foals and offer new perspectives for diagnosing and treatment ALDs in newborn foals.

## 1 Introduction

Angular limb deformities (ALDs) are common skeletal deformities in horses, manifesting as valgus or varus deformities, often accompanied by some axial rotation (1). Initially, these deformities are postural. However, prolonged external pressure on the bone can increase bone density and hardness over time. The uneven growth of the epiphyseal growth plate can lead to permanent angulation deformities (2). Therefore, early detection and intervention are crucial to prevent these temporary postural changes from developing into permanent structural deformities.

The causes of ALDs are complex and can be summarized into perinatal and developmental factors (3). Perinatal factors include adverse effects on the foal during late pregnancy and delivery. Developmental factors encompass various influences causing limb axis abnormalities in foals during growth. These influencing factors are closely related to the nutritional status of the mare, leading to changes in conventional blood markers such as blood physiological and biochemical indicators, enzymes and metabolites, thus affecting the growth and development and nutritional metabolism of the offspring (4, 5). Intestinal flora plays a key role in the early growth and development of horses, promoting the proliferation of intestinal cells and providing energy by decomposing cellulose to produce short-chain fatty acids such as propionic acid, acetic acid, and butyric acid (6). Intestinal microbes help build an immune barrier to resist the invasion of foreign pathogens by activating immune cells under the host's intestinal mucosa. Specific intestinal microbes can also promote the formation of immune tolerance and reduce the risk of allergic and autoimmune diseases (7). Additionally, intestinal flora is crucial for bone growth, development, and metabolism by regulating the absorption of essential nutrients like calcium and phosphorus (8). For example, it regulates intestinal pH levels, optimizes the depth ratio of intestinal villi to crypts, and inhibits programmed cell death of epithelial cells, ensuring effective absorption of key nutrients such as calcium and phosphorus (9). Studies have found an association between gut microbiota and the development of inflammatory bowel diseases, which may interfere with the intestinal absorption of calcium and vitamin D, indirectly affecting bone tissue metabolism and increasing the risk of bone health issues (10). These all indicate that intestinal flora is closely related to inflammatory and metabolic diseases of the host, but it is not clear whether the intestinal flora of newborn foals with ALDs and their mares are different from those of normal foals and their mares.

Therefore, this paper intends to investigate ALDs and then explore the differences in blood physiological and biochemical indicators and intestinal flora between ALDs foals and their mares and healthy newborn foals and their mares, in order to clarify the relationship between the occurrence and development of ALDs and intestinal flora and provide new insights into the prevention and treatment of ALDs.

## 2 Materials and methods

### 2.1 Experimental animals

This study investigated the epidemiology of ALDs in 460 newborn foals of Ili horses, purebred horses, and crossbred horses (Foals, 2–8 days old, mares, 5–14 years old) in the Zhaosu area (Xinjiang, China) from April to June 2023. The study included foals of both genders. The horse owners and stables approved all investigations, and informed consent was signed. The Animal Welfare and Ethics Committee of Xinjiang Agricultural University approved the experimental design and animal ethics (2023033).

### 2.2 ALDs prevalence survey

#### 2.2.1 Diagnosis

ALDs was diagnosed using morphological observation and clinical portable X-ray imaging (YZB0671-2007, Dandong Keda Instrument Co., Ltd., China). This combined approach allowed for the accurate determination of deformity types and severity and the evaluation of bone symmetry and joint integrity.

#### 2.2.2 Morphological examination

Clinical observations were first conducted on the newborn foals. The foal was positioned on a flat surface and observed by a veterinarian from the front, sides, and back, with particular attention to the morphological symmetry of the wrist and tarsal joints. The foal was driven to walk slowly and quickly for gait examination to observe limb coordination and claudication. Suspected morphologically abnormal joints were then examined in detail. The veterinarian held the metacarpal bones and attempted to gently bend the wrist or tarsus to be parallel to the radius or tibia. Finally, the site of the deformity was evaluated by comparing it with the contralateral limb. Clinical observations were performed by two veterinarians blinded of the study details.

#### 2.2.3 Imaging examination

In foals with suspected limb deformities identified during morphological examination, X-ray imaging was performed to confirm ALDs. The primary projection positions for imaging were dorsolateral and dorsomedial. The X-ray was centrally aligned to examine the middle of the joint, including the long bones above and below the joints. On the X-ray film, two straight lines were drawn from the long bones above and below the joint along their long axes. The intersection point of these lines indicated the location of the deformity, and the angle formed represented the degree of deviation (indicating the severity of the deformity).

### 2.3 Blood physiological and biochemical indicators and intestinal microbiome detection

#### 2.3.1 Sample collection

Horses were divided into four groups: healthy foals, healthy mares, ALDs foals, and ALDs mares. Five milliliter of blood was collected through the jugular vein using a vacuum blood collection needle for determination of blood physiological and biochemical indicators of the foals. Fresh fecal samples from each group were collected using sterile cotton swabs (pre-moistened with normal saline) that were slowly inserted 2–3 cm into the horses' anus. The samples were placed into sterile frozen storage tubes and stored in liquid nitrogen until analysis.

#### 2.3.2 Blood physiological and biochemical indices detection

Blood physiological and biochemical parameters of normal foals and ALDs foals and normal mares and ALDs mares were measured using BC-5300Vet Mindray fully automatic five-category animal blood cell analyzer and BS-240VET Mindray fully automatic biochemical analyzer (Shenzhen, China), respectively.

#### 2.3.3 DNA extraction and PCR amplification

DNA was extracted using the CTAB method (Phusion^®^ High-Fidelity DNA polymerase, New England Biolabs, USA). The purity and concentration of the DNA were then measured. The V3 + V4 variable region of the DNA was amplified by PCR using specific primers (Table 1).

**Table 1:** Primer information.

**Primer**	**Primer sequence**	**Product length/bp**
341F	5′-CCTAYGGGRBGCASCAG-3′	468
806R	5′-GGACTACNNGGGTATCTAAT-3′	

#### 2.3.4 Purification and mixing of PCR products

Aliquots were mixed based on concentration. After thorough mixing, the products were purified using 2% agarose gel electrophoresis. The universal DNA purification recovery kit (TianGen, China) was used to recycle the bands of interest.

#### 2.3.5 Library construction and sequencing

The NEB Next^®^ Ultra DNA Library Prep Kit (New England Biolabs) was used for library construction. The quality of the prepared libraries was checked and quantified using the Agilent 5400 Fragment Analyzer (USA). After confirming the library met the quality standards, high-throughput sequencing was performed using the Illumina platform.

Potential contaminating sequences, such as mitochondrial and chloroplast sequences, were excluded using the Feature-table plugin of QIIME2 to ensure result accuracy. Differences in the abundance of the microbiota among the different samples were identified using two statistical methods: analysis of variance (ANOVA) and linear discriminant analysis effect size (LefSe). Correlation matrices for alpha diversity and beta diversity were calculated using the core-diversity plugin of QIIME2.

### 2.4 Data processing and statistical analysis

Statistical analyses were performed using statistical package for social sciences (SPSS) software (version 19.0 for Windows, SPSS, Chicago, IL, United States). The results are presented as mean ± standard deviation. For two-group comparisons, data were analyzed using a two-tailed Student's *t*-test. For multiple group comparisons, statistical analysis was performed using ANOVA followed by the least significant difference test. *P* < 0.05 was considered statistically significant.

## 3 Results

### 3.1 Typical foals with ALDs

**Figure 1:**
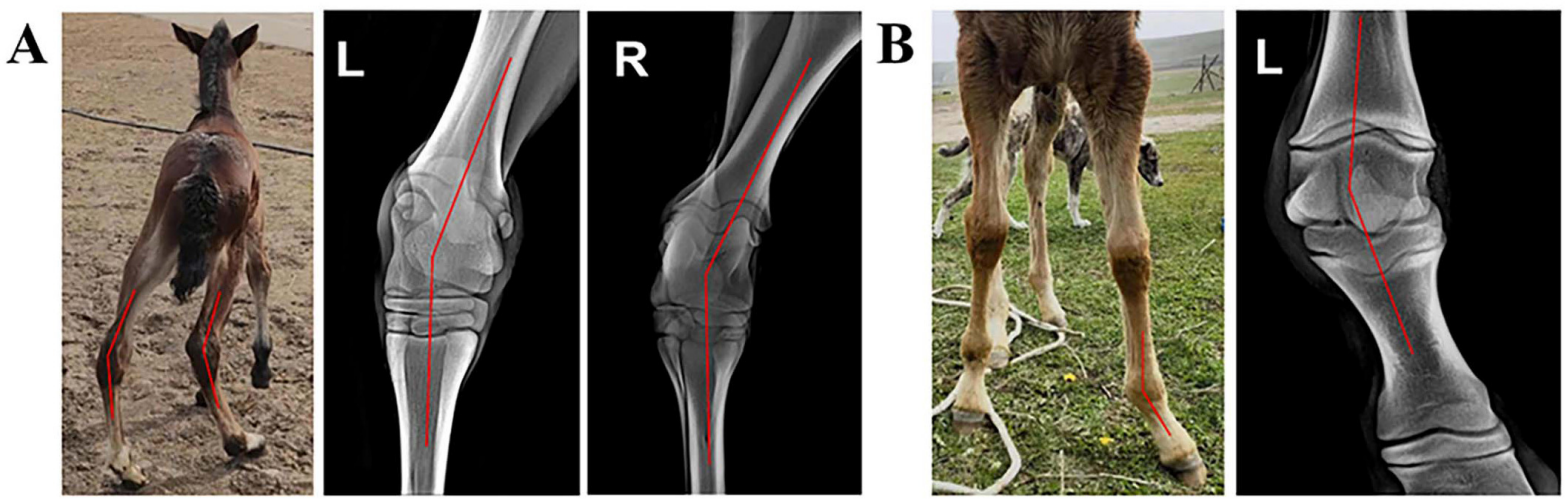
Deformities of different foals. **(A)** A 14-day-old purebred male foal with a valgus deformity of the left forelimb fetlock. The dorsal view of the foal is shown in the middle, and dorsal metacarpal radiographs of the tarsal joint area of both hind limbs are on the left and right. **(B)** A 3-day-old male crossbred foal with a windswept deformity. The frontal view of the foal is on the left, and the dorsal metacarpal radiograph of the fetlock area of the left forelimb is on the right.

Morphological examination revealed that Foal A, a 3-day-old male crossbred foal (its mare was a 9-year-old crossbred horse that had given birth five times), had bilateral tarsal distortion (Figure 1A). Foal B, a 14-day-old male purebred foal (its mare was a 12-year-old purebred horse that had given birth nine times), had lateral fetlock distortion on the left forelimb (Figure 1B). X-ray imaging diagnosed Foal A with varus of the left hind limb tarsal joint and valgus of the right hind limb tarsal joint, indicating a windswept deformity (Figure 1A). Foal B was diagnosed with a valgus deformity of the left forelimb fetlock (Figure 1B).

### 3.2 The influence of different factors on the prevalence of ALDs

**Figure 2:**
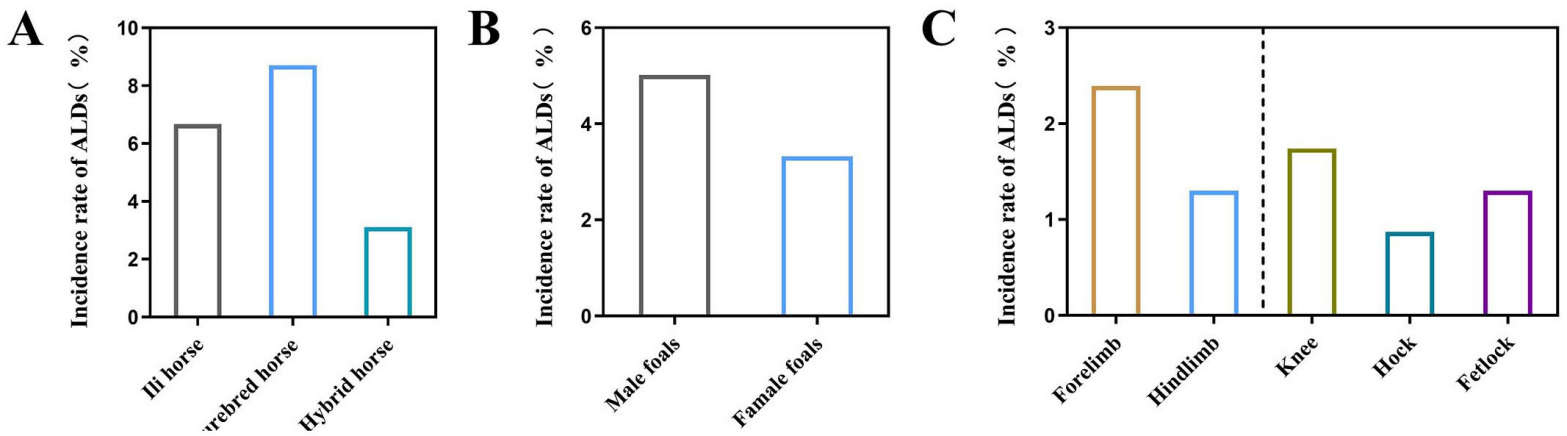
Influence of different factors on the prevalence of ALDs in foals. **(A)** The incidence of ALDs in different breeds of foals. **(B)** The incidence of ALDs in foals of different sexes. **(C)** The incidence of ALDs in foals with different malformed parts.

Among the 460 newborn foals surveyed, 19 were diagnosed with ALDs (4.13%). The incidence in various breeds was found to be 6.67% (4/60) in Ili horses, 8.70% (4/46) in purebred horses, and 3.11% (11/354) in hybrid horses (Figure 2A). The results showed that 5.02% (11/219) of male foals and 3.32% (8/241) of female foals were diagnosed with ALDs (Figure 2B). Among the 19 diagnosed ALD foals, the deformities involved the forelimbs and hindlimbs, specifically the wrist joint, tarsal joint, and fetlock. The proportion of forelimb deformities was 2.39% (10/460), while hindlimb deformities was 1.30% (6/460). Wrist deformities accounted for 1.74% (7/460), tarsal joint deformities for 0.87% (4/460), and fetlock deformities for 1.3% (5/460) (Figure 2C).

### 3.3 Differential blood physiological and biochemical indices of ALDs

**Figure 3:**
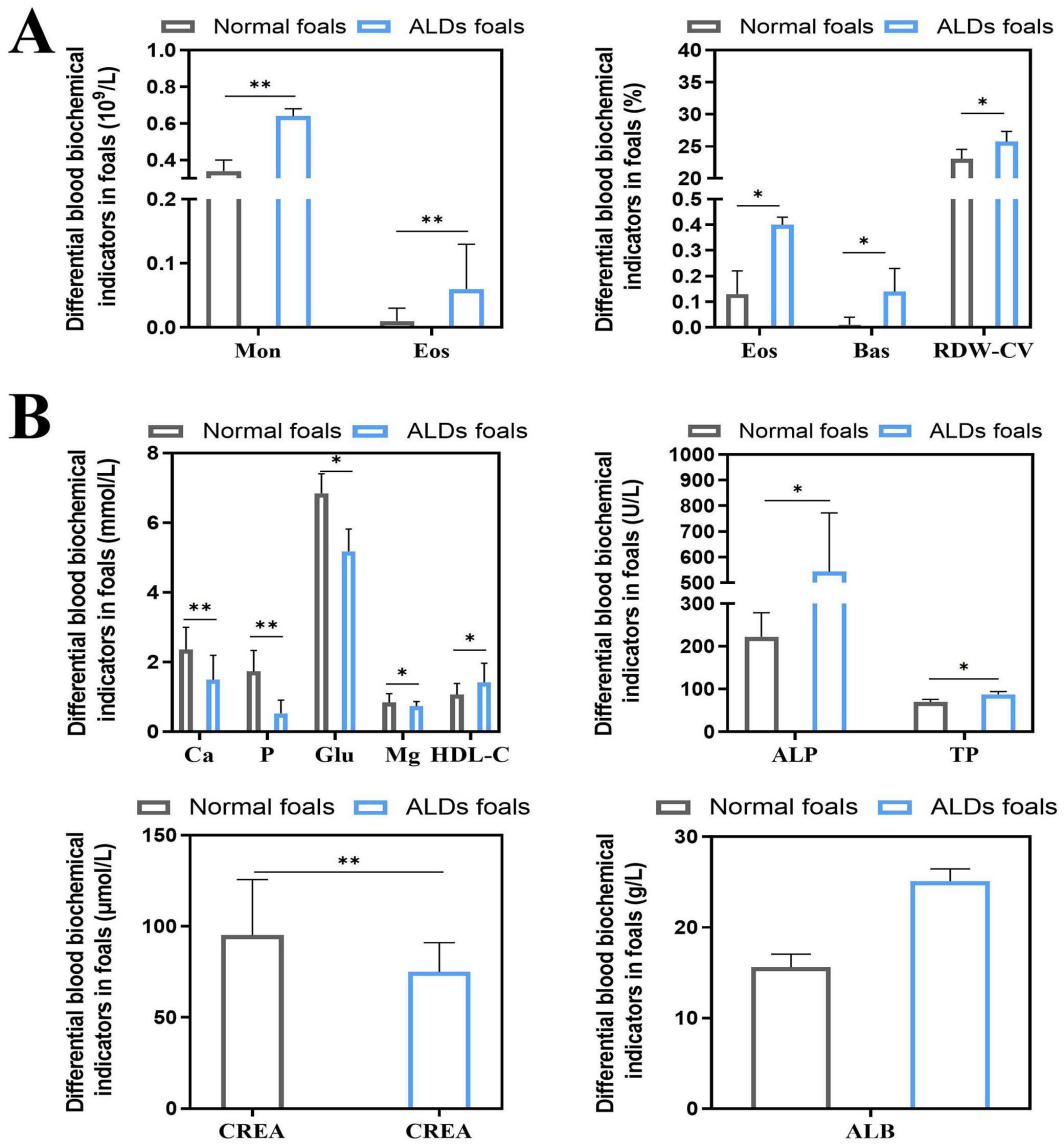
Differential changes in blood indices between ALDs foals. **(A)** Differential changes in blood physiological parameters in ALDs foals. **(B)** Differences in blood biochemical parameters in ALDs foals. *P* < 0.05 was considered statistically significant. ^*^P < 0.05; ^**^P < 0.01.

The results of blood physiological indices of normal foals and ALDs foals are shown in Figure 3A. Monocyte (Mon), Eosinophil (Eos), Eosinophil% (Eos%), Basophil% (Bas%), and Erythrocyte distribution width CV (RDW-CV) of ALDs foals were significantly higher than those of normal foals (*P* < 0.05). The results of blood biochemical indexes of normal foals and ALDs foals are shown in Figure 3B. The levels of Calcium (Ca), Phosphorus (P), Glucose (Glu), and Creatinine (CREA) of ALDs foals were significantly lower than those of normal foals (*P* < 0.05), and the levels of High density lipoprotein cholesterol (HDL-C), Alkaline phosphatase (ALP), Total protein (TP), and Albumin (ALB) of ALDs foals were significantly higher than those of normal foals (*P* < 0.05).

### 3.4 Hormone and inflammatory cytokine levels in ALDs foals

**Figure 4:**
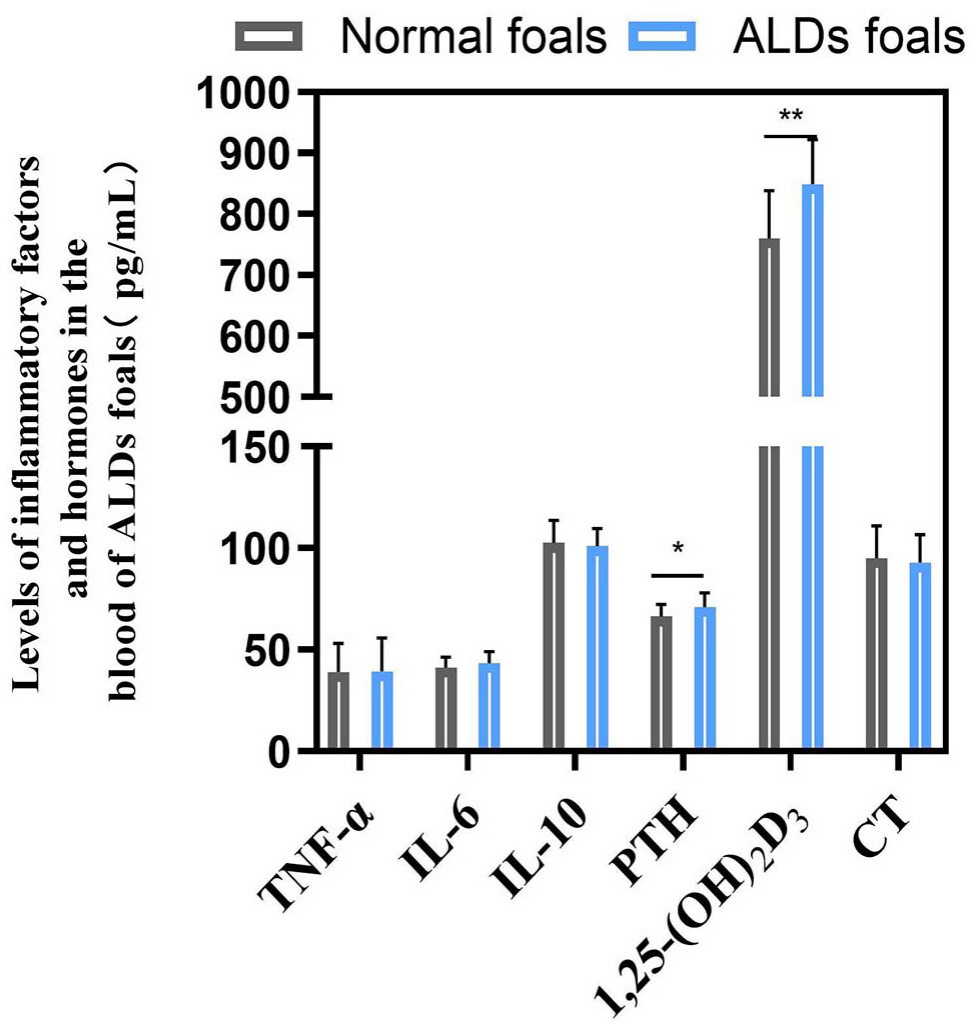
Blood inflammatory factors and hormone levels in ALDs foals. *P* < 0.05 was considered statistically significant. ^*^P < 0.05; ^**^P < 0.01.

There was no significant change in blood inflammatory factors between ALDs foals and normal foals (*P* > 0.05). The levels of PTH and 1,25-(OH)2D3 in ALDs foals were significantly or extremely significantly higher than those in normal foals (*P* < 0.05) (Figure 4).

### 3.5 Intestinal flora diversity and LefSe analysis

**Figure 5:**
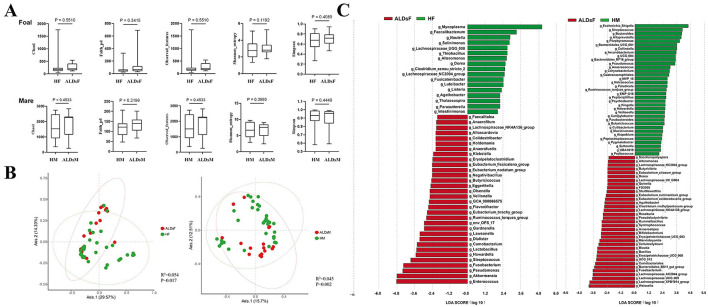
LefSe analysis of gut microbiota. **(A)** Comparison of the alpha diversity index of the intestinal flora in normal and ALDs foals (top) and in normal and ALDs mares (bottom). **(B)** Principal coordinate analysis of normal foals and ALDs foals (left), as well as normal mares and ALDs mares (right) based on Bray-Curtis distance. **(C)** LDA plots of LefSe analysis of normal foals and ALDs foals (left) and normal mares and ALDs mares (right). HF, healthy foals; ALDsF, ALDs foals; HM, healthy mares; ALDsM, ALDs mares.

The Chao1, Faith_pd, Observed_features, Shannon_entropy, and Simpson index of the intestinal flora of normal foals and ALDs foals, as well as normal mares and ALDs mares, were analyzed. No significant differences were found between the two groups of normal foals and ALD foals, neither between the two groups of normal mares and ALD mares (*P* > 0.05) (Figure 5A).

Bray-Curtis distance analysis was used to perform principal coordinates analysis comparative analysis on the intestinal flora structure of normal foals, ALDs foals, and normal mares and ALDs mares. The results showed that samples within normal and ALD foals groups clustered together while the groups were separated. An inter-group Anosim test revealed that the composition of the intestinal flora of normal foals and ALD foals was significantly different (*R*^2^ = 0.054, *P* = 0.017). The samples from normal mares and ALDs mares were clustered together, and the groups were obviously separated. The inter-group Anosim test showed that the intestinal flora composition of normal mares and ALDs mares was extremely significantly different (*R*^2^ = 0.045, *P* = 0.002) (Figure 5B).

LefSe analysis results indicated that the absolute value of LDA was log ≥ 1.0. In normal foals, there were 32 dominant bacterial groups at the genus level (such as *Faecalitalea, Anaerofilum*, and *Lachnospiraceae*_NK4A136_group), and 17 in ALDs foals (e.g., *Mycoplasma, Faecalibacterium*, and *Nautilia*). In normal mares, the dominant bacterial flora consisted of 30 genera (such as *Saccharopolyspora, Alteromonas*, and *Lachnospiraceae_*NC2004_group), while in ALDs mares, it consisted of 23 genera (such as *Escherichia_Shigella, Streptococcus*, and *Bacteroides*). This suggests clear differences in the gut microbiome composition between normal and ALD status (Figure 5C).

### 3.6 Analysis of the composition and relative abundance of intestinal flora

**Figure 6:**
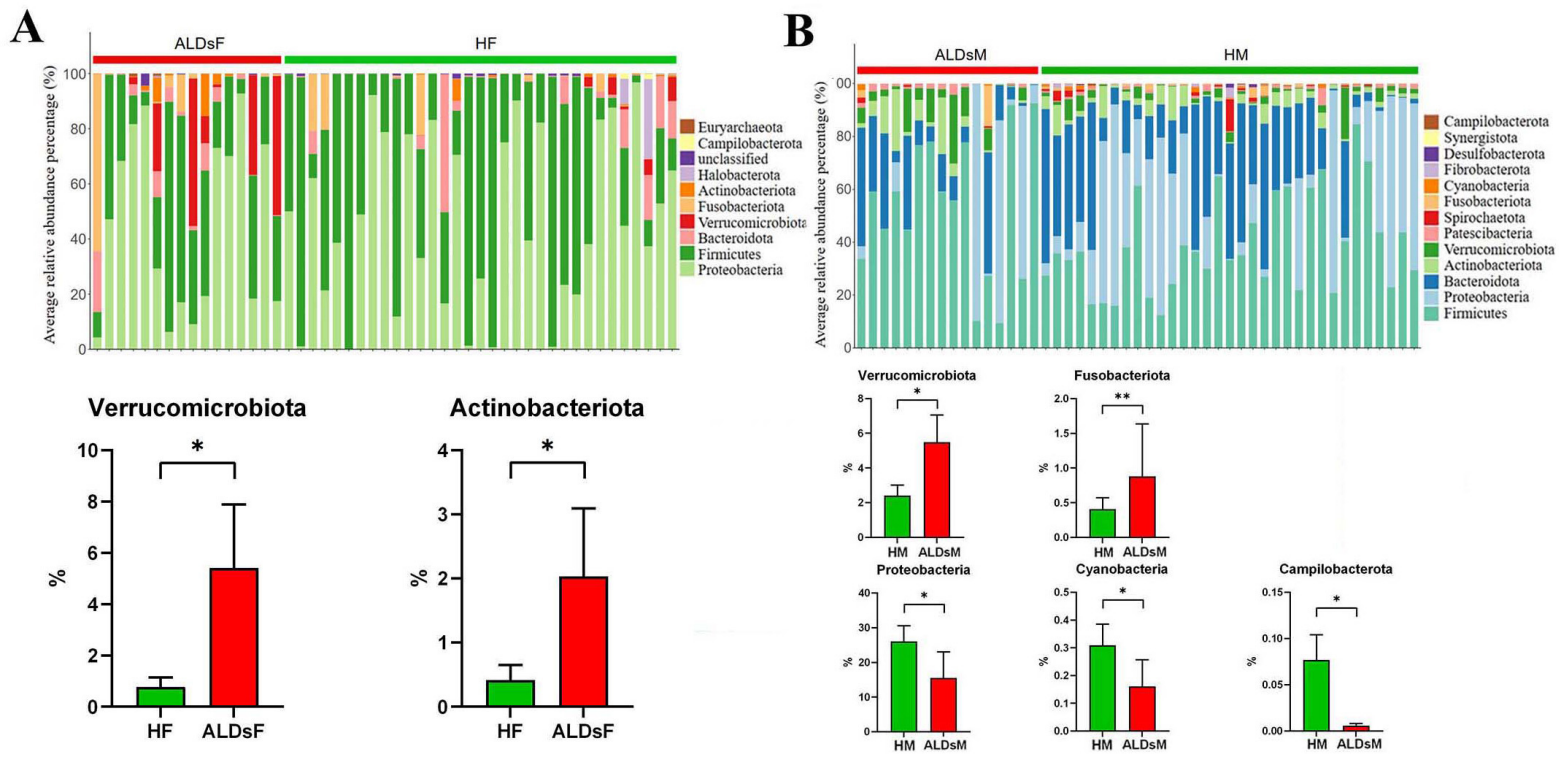
Analysis of microphyla composition and relative abundance of gut microbiota. **(A)** Bacterial phyla with an average relative abundance > 0.1% in normal and ALDs foals (above). Microphyla with significantly increased relative abundance in ALDs foals (below). **(B)** Bacterial phyla have an average relative abundance > 0.1% in normal and ALDs mares (above). Microphyla has significant increases and decreases in relative abundance in ALDs mares (below). HF, healthy foals; ALDsF, ALDs foals; HM, healthy mares; ALDsM, ALDs mares. *P* < 0.05 was considered statistically significant. ^*^P < 0.05; ^**^P < 0.01.

The top 0.1% of the average relative abundance of intestinal flora was selected for further analysis. Among foals, the dominant phyla were *Proteobacteria, Firmicutes, Bacteroidota, Verrucomicrobiota*, and *Fusobacteriota*, with others including *Actinobacteriota, Halobacterota*, unclassified, *Campilobacterota*, and *Euryarchaeota* (Figure 6A). In mares, the dominant phyla were *Firmicutes, Proteobacteria, Bacteroidota, Actinobacteriota, Verrucomicrobiota*, and *Patescibacteria*, with others including *Spirochaetota, Fusobacteriota, Cyanobacteria, Fibrobacterota*, Desulfobacterota, *Synergistota*, and *Campilobacterota* (Figure 6B).

Using an independent sample *T*-test, the relative abundance of *Verrucomicrobiota* and *Actinobacteriota* in the intestinal flora of ALD foals was significantly increased compared to normal foals (*P* < 0.05) (Figure 6A). In ALD mares, the relative abundance of *Verrucomicrobiota* and *Fusobacteriota* was significantly increased (*P* < 0.05). In contrast, the relative abundance of *Campylobacterota, Cyanobacteria*, and *Proteobacteria* was significantly decreased (*P* < 0.05) compared to normal mares (Figure 6B).

### 3.7 Kyoto encyclopedia of genes and genomics function prediction of intestinal flora and differential analysis of three-level metabolic functions

**Figure 7:**
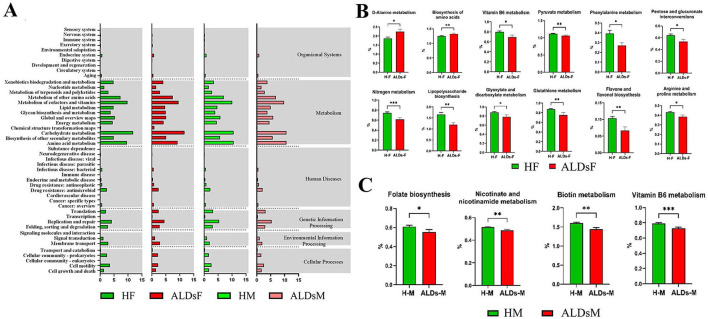
KEGG function of gut microbiota and differential analysis of tertiary metabolic function. **(A)** KEGG function prediction. **(B)** Comparison of metabolic functions of gut microbiota in normal foals and ALDs foals. **(C)** Comparison of metabolic functions of intestinal flora in normal mares and ALDs mares. HF, healthy foals; ALDsF, ALDs foals; HM, healthy mares; ALDsM, ALDs mares. *P* < 0.05 was considered statistically significant. ^*^P < 0.05; ^**^P < 0.01; ^***^P < 0.001.

KEGG function prediction of intestinal flora showed six biological metabolic functional pathways at the first-level functional level. The highest average relative abundance was metabolism at 71.8%, followed by genetic information processing at 10.0%, cellular processes at 6.2%, human diseases at 5.7%, environmental information processing at 4.1%, and organic systems at 2.3%. At KEGG level 2, a total of 47 metabolic pathways were detected. The top 10 in terms of average relative abundance were carbohydrate metabolism (11.7%), cofactors and vitamins metabolism (9.6%), amino acid metabolism (9.2%), other amino acids metabolism (7.5%), other secondary metabolites biosynthesis (5.1%), global and overview maps (5.1%), glucose biosynthesis and metabolism (5.0%), lipid metabolism (4.8%), replication and repair (4.5%), and energy metabolism (4.2%) (Figure 7A).

Based on KEGG level 3 analysis, 12 metabolic pathways showed differences. In ALD foals, the relative abundance of D-alanine metabolism and biosynthesis of amino acids pathways was significantly increased (*P* < 0.05) compared to normal foals. In contrast, the relative abundance of vitamin B6 metabolism, pyruvate metabolism, phenylalanine metabolism, pentose and glucuronate interconversions, nitrogen metabolism, lipopolysaccharide biosynthesis, glyoxylate and dicarboxylate metabolism, glutathione metabolism, flavone and flavonol biosynthesis, and arginine and proline metabolism pathways was significantly decreased (*P* < 0.05) (Figure 7B). In ALD mares, four differential metabolic pathways were identified (Figure 7C). Compared to normal mares, the relative abundance of biotin metabolism, vitamin B6 metabolism, folate biosynthesis, and nicotininate and nicotinamide metabolism pathways were significantly decreased (*P* < 0.05).

### 3.8 Correlation analysis of differential physiological and biochemical indicators, intestinal bacteria genera and KEGG differential functions

**Figure 8:**
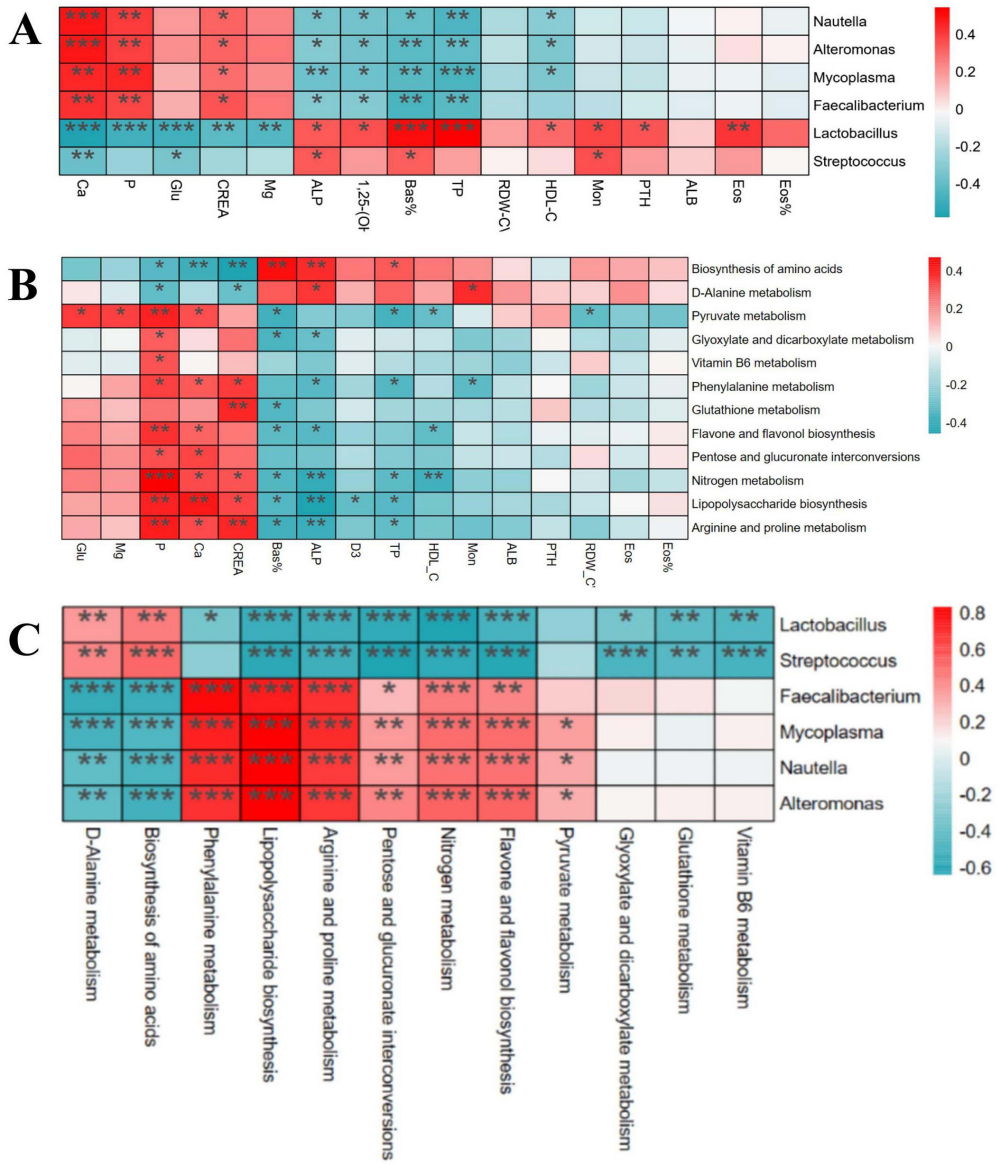
Correlation analysis of differential physiological and biochemical indexes, intestinal bacteria genera and KEGG differential function in foals. **(A)** Correlation analysis between different strains and different physiological and biochemical indexes. **(B)** Correlation analysis between different physiological and biochemical indexes and KEGG tertiary metabolic function. **(C)** Correlation analysis between different strains and KEGG tertiary metabolic function.

Through Spearman correlation analysis, the results showed that there were multiple significant correlations between the physiological and biochemical indicators of foals (Figure 8A) and the differential bacterial genera [(*P* < 0.05), red indicates positive correlation, blue indicates negative correlation]. There was a significant correlation between the differential physiological and biochemical indicators of foals (Figure 8B) and the KEGG tertiary metabolic functions [(*P* < 0.05), red indicates positive correlation, blue indicates negative correlation]. Interestingly, there was also a significant correlation between the differential bacterial genera of foals (Figure 8C) and the KEGG tertiary metabolic functions [(*P* < 0.05), red indicates positive correlation, blue indicates negative correlation].

## 4 Discussion

ALDs are common bone development disorders in newborn foals, significantly impacting their future athletic performance and economic value. Inflammations during pregnancy, such as placentitis and metritis, as well as severe metabolic diseases, are significant causes of ALDs in newborn foals (11). The intestinal flora is closely linked to host health and disease (12). This study first investigated the current status of ALDs in newborn foals and, based on these findings, evaluated the changes in blood physiological and biochemical parameters of ALDs foals, used 16sRNA gene sequencing technology to analyze changes in the composition and function of their intestinal flora to reveal the relationship between intestinal flora and ALDs.

A survey of 460 newborn foals revealed that 19 foals (4.13%) were diagnosed with ALDs. Analyzing the incidence of ALDs among different breeds showed that purebred horses had the highest incidence (8.70%), followed by Ili horses (6.67%), with hybrid horses having a relatively low incidence (3.11%). Studies have shown that the incidence of ALDs in purebred horse populations can be as high as 11% (12). These findings suggest that horse breeds have varying susceptibility to ALDs, likely related to genetic factors. Due to long-term selective breeding, purebred horses may have accumulated more genetic variants that cause ALDs (13). In contrast, hybrid horses benefit from diverse genetic backgrounds, which reduces the risk of certain genetic diseases (14). These data emphasize the importance of personalized management and prevention. Purebred horses require more sophisticated management and monitoring, but hybrid horses should not be overlooked. Gender also influences the incidence of ALDs. The incidence rate in males (5.02%) is higher than in females (3.32%), which may be related to genetic factors, hormone levels, and metabolic processes (15). Genes on sex chromosomes (such as X and Y chromosomes) are associated with sex-specific disease susceptibility (16). If ALDs are related to genes on the Y chromosome, males (XY) and females (XX) would have different genetic susceptibilities (17). Additionally, male and female foals experience various levels of exercise, nutrition, and other management methods, indirectly affecting the development of ALDs. Studies have shown that male foals exercise more frequently in their early years, impacting limb development (18). The location of limb deformities is also related to the prevalence of ALDs. Results showed a high incidence of forelimb deformities (2.39%), with wrist deformities being the most common (1.74%). This may be related to the physiological structure and movement pattern of horses. When horses exercise, their forelimbs bear the most weight and impact, making them more deformable (19). Additionally, the anatomical structure of the forelimbs, such as the position and function of the wrist joint, is more vulnerable (20).

ALDs can be diagnosed based on clinical observation and imaging changes. The purpose of testing the physiological and biochemical indicators of ALD foals is to assist in observing the body state of ALD foals through changes in these indicators, so as to evaluate the physiological function and immune function of the foals. In this study, the white blood cell indexes of ALDs foals, such as Mon, Eos, Eos%, and Bas, were significantly higher than those of normal foals. This result suggests that ALDs foals induce stress response due to limb abnormalities. GLU in the blood is essential for the synthesis of non-essential amino acids, which are further involved in the process of protein synthesis. In this study, we found that the GLU level of ALDs newborn foals was significantly lower than that of normal foals, indicating that the energy and nutritional level of ALDs newborn foals was insufficient. TP and ALB levels reflect the protein synthesis capacity of the liver and the maintenance of plasma osmotic pressure, and are important indicators of animal liver function and energy metabolism. ALP elevation is associated with changes in liver function and abnormal bone growth. The ALP level of ALDs foals was significantly higher than that of normal foals, indicating that ALDs foals had abnormal bone development. The role of calcium and phosphorus in bone formation and bone growth and development cannot be ignored. The serum Ca and P content of LDs newborn foals was significantly lower than that of normal foals, and the Ca and P levels were lower than the normal reference range, indicating that the deficiency of these mineral elements may directly affect the bone growth and development of foals. Combined with the above experimental results, this reflects that ALDs foals are suffering from malnutrition and mineral deficiency, which is speculated to be related to the occurrence of ALDs in foals. Although the use of hematological indicators alone cannot provide a specific early diagnosis of ALDs, changes in hematological indicators of ALDs foals can reflect the nutritional status of foals in areas with high incidence of ALDs. By judging the changes in the body state of ALDs foals, ALDs can be prevented and treated from multiple aspects and angles, providing a theoretical reference for nutritional supplementation and healthy breeding of newborn foals.

The intestinal flora comprises a complex ecosystem of microorganisms that convert nutrients entering the intestine into essential substances for normal bodily growth and development (21). It is crucial in maintaining normal digestion, nutrient absorption, energy conversion, and animal metabolic functions (22). In recent years, more and more attention has been paid to the effect of intestinal flora on animal bone development and its mechanism. Many studies have demonstrated the role of intestinal flora in nutrient absorption, immunity, endocrine function and metabolite production, especially on bone metabolism, providing a new way to prevent and treat animal bone diseases (23). Regarding the alpha diversity index, encompassing Chao1, Faith_pd, Observed_features, Shannon_entropy, and Simpson index, no significant differences were found between normal foals and those with ALDs, nor between normal mares and ALDs mares. This indicates comparable richness and uniformity in intestinal flora among the four groups at the alpha diversity level. Beta diversity, a critical indicator for assessing microbial composition differences across samples and communities, revealed distinct intestinal microbiota structures between ALD and normal foals and between normal mares and ALD mares (24). While the total count and distribution of microbiota were similar, specific microbial abundances differed between normal and ALD states, suggesting a potential link between intestinal microbiota and ALD occurrence. LefSe analysis identified 17 dominant microbiota in ALD foals, including mycoplasma, known to induce various diseases in animals and humans (25). Mycoplasma's presence may indirectly impact bone development by triggering chronic inflammation or affecting the host's immune response (26). *Faecalibacterium prausnitzii*, a beneficial bacterium within the *Faecalibacterium* genus, is associated with anti-inflammatory effects (27). This suggests a protective role in combating ALD-related inflammationby increasing anti-inflammatory microbiota (28). In ALD mares, 23 dominant bacterial groups were identified, such as *Escherichia_Shigella* and *Streptococcus*, known pathogens capable of causing intestinal and systemic infections (29). Their dominance in ALD mares may correlate with their health status (30). Notably, ALD foals and mares exhibited more pathogenic bacteria in their intestines than their normal counterparts. *Streptococcus*, uncommon in normal intestinal flora, may play a significant role in the unique intestinal environment of ALD mares, potentially promoting ALD development. This similarity in the intestinal environment between ALD foals and mares suggests a potential influence of genetic or environmental factors on the occurrence of ALDs.

Further analysis of the phylum structure of the intestinal flora revealed that in both normal and ALD foals, the average relative abundance of *Proteobacteria, Firmicutes, Bacteroidota, Verrucomicrobiota*, and *Fusobacteriota* exceeded 1%, classifying them as high-abundance phyla. Similarly, in normal and ALDs mares, *Firmicutes, Proteobacteria, Bacteroidota, Actinobacteriota, Verrucomicrobiota*, and *Patescibacteria* were high-abundance phyla. Notably, the abundance of *Verrucomicrobiota* and *Actinobacteriota* increased significantly in ALD foals. *Akkermansia muciniphila*, a bacterium in the phylum *Verrucomicrobia*, enhances intestinal barrier function, modulates immune responses, and is associated with reduced inflammation (31). The phylum *Actinobacteriota* includes many antibiotic-producing bacteria, such as *Bifidobacterium*, which can modulate the immune system and suppress inflammation (32). The increase in these bacteria in ALD foals suggests that the intestinal microbiome responds to the disease state by increasing beneficial bacteria to alleviate inflammation or mucosal damage caused by ALDs. Conversely, the phylum *Fusobacteria*, associated with various inflammatory diseases, increased, indicating an aggravated intestinal inflammatory state in ALD mares and a potential link to ALDs in foals (33).

Numerous recent studies have increasingly revealed the intricate link between intestinal microorganisms and various diseases (34). Particularly, the “gut-bone axis” concept underscores the complex interaction between intestinal flora and bone health. For example, intestinal flora regulates bone metabolism and health by influencing nutrient absorption, immune regulation, and hormone levels through its metabolic activities (23). Microbial metabolites, such as short-chain fatty acids (SCFAs), have positively impacted bone health by promoting calcium absorption and increasing bone density (35). Moreover, microorganisms can mitigate bone health issues by reducing inflammatory responses (36). These studies all demonstrate a close link between gut microbiota and bone metabolism. The results show that a total of 12 metabolic pathways were found to significantly differ between ALD and normal foals. Among these pathways, many can affect bone metabolism. D-alanine metabolism regulates bone energy metabolism by modulating insulin secretion and participating in blood glucose regulation in mammals (37). Abnormal nitrogen metabolism and the interconversion pathways of pentose and glucuronic acid, closely tied to energy metabolism, affect protein synthesis and degradation, thereby influencing bone health (37). Additionally, the downregulation of glutathione metabolism can induce oxidative stress, pivotal in the pathogenesis of growth and developmental diseases (38). These results are consistent with previous studies highlighting the critical role of the gut microbiome in host immune and metabolic processes, which directly affect bone development and health (39, 40). Similarly, ALD mares exhibit significantly distinct metabolic pathways compared to normal mares, particularly in biotin metabolism, vitamin B6 metabolism, folate biosynthesis, niacin, and nicotinamide metabolism. Biotin metabolism, crucial for synthesizing and degrading key biological molecules, influences fatty acid synthesis and amino acid metabolism, essential for normal bone development (41). Dysfunction in the folate biosynthesis pathway, essential for energy metabolism, can lead to various health issues, including congenital disabilities and osteoporosis (42). Vitamin B6 metabolism affects intestinal inflammation levels and the host immune response, which is critical for maintaining normal metabolism, especially anti-inflammatory responses (43). Niacin and nicotinamide metabolism are linked to energy metabolism and DNA repair, which are vital for maintaining bone cell health and function (44). Further, through correlation analysis, we found that *Lactobacillus* was negatively correlated with Ca, P, and Glu indices in ALDs foals, and was positively correlated with 1,25-(OH)2D3 and PTH. *Faecalibacterium* was positively correlated with Ca, P, and Glu indices, and negatively correlated with 1,25-(OH)2D3 and PTH. *Lactobacillus* and *Streptococcus* were positively correlated with Biosynthesis of amino acids, negatively correlated with Arginine and proline metabolism, and negatively correlated with Glutathione metabolism. This indicates that intestinal flora imbalance in foals is associated with the physiological and biochemical indicators of ALDs foals. These results suggest that mares may mediate skeletal development in foals by modulating their gut microbiota to influence their own nutrient absorption, immune regulation, and metabolite production. Therefore, it is speculated that gut microbiota may be involved in the pathogenesis of ALDs through its role in the “gut-bone axis”.

In summary, blood markers such as calcium, phosphorus, and blood glucose were altered in ALDs foals, and the diversity, species composition, and function of the intestinal flora of ALDs and their mares were significantly altered. Compared with ALD foals, normal foals showed significant differences in *Lactobacillus* and *Faecalibacterium*, which could become potential markers of ALDs. Compared with ALD mares, normal mares showed significant differences in *Escherichia-Shigella* and *Pseudomonas*, which could become potential markers of ALDs. We speculate that there may be a pathogenic mechanism of ALDs in foals through the “blood marker changes-gut microbiota-ALDs” axis, but further mechanistic studies are needed in the future. In conclusion, these findings provide new insights into the interaction between gut microbiota and bone health and provide possible targets for the development of early diagnosis techniques and prevention and treatment strategies.

## Data Availability

The original contributions presented in the study are included in the article/supplementary material, further inquiries can be directed to the corresponding authors.
